# Poultry Coccidiosis: Design and Interpretation of Vaccine Studies

**DOI:** 10.3389/fvets.2020.00101

**Published:** 2020-02-26

**Authors:** Francesca Soutter, Dirk Werling, Fiona M. Tomley, Damer P. Blake

**Affiliations:** Department of Pathobiology and Population Sciences, Royal Veterinary College, Hertfordshire, United Kingdom

**Keywords:** chickens, *Eimeria*, coccidiosis, vaccine development, *in vivo* trials

## Abstract

*Eimeria* infection impacts upon chicken welfare and economic productivity of the poultry sector. Live coccidiosis vaccines for chickens have been available for almost 70 years, but the requirement to formulate blends of oocysts from multiple *Eimeria* species makes vaccine production costly and logistically demanding. A multivalent vaccine that does not require chickens for its production and can induce protection against multiple *Eimeria* species is highly desirable. However, despite the identification and testing of many vaccine candidate antigens, no recombinant coccidiosis vaccine has been developed commercially. Currently, assessment of vaccine efficacy against *Eimeria*, and the disease coccidiosis, can be done only through *in vivo* vaccination and challenge experiments but the design of such studies has been highly variable. Lack of a “standard” protocol for assessing vaccine efficacy makes comparative evaluations very difficult, complicating vaccine development, and validation. The formulation and schedule of vaccination, the breed of chicken and choice of husbandry system, the species, strain, magnitude, and timing of delivery of the parasite challenge, and the parameters used to assess vaccine efficacy all influence the outcomes of experimental trials. In natural *Eimeria* infections, the induction of strong cell mediated immune responses are central to the development of protective immunity against coccidiosis. Antibodies are generally regarded to be of lesser importance. Unfortunately, there are no specific immunological assays that can accurately predict how well a vaccine will protect against coccidiosis (i.e., no “correlates of protection”). Thus, experimental vaccine studies rely on assessing a variety of post-challenge parameters, including assessment of pathognomonic lesions, measurements of parasite replication such as oocyst output or quantification of *Eimeria* genomes, and/or measurements of productivity such as body weight gain and feed conversion rates. Understanding immune responses to primary and secondary infection can inform on the most appropriate immunological assays. The discovery of new antigens for different *Eimeria* species and the development of new methods of vaccine antigen delivery necessitates a more considered approach to assessment of novel vaccines with robust, repeatable study design. Careful consideration of performance and welfare factors that are genuinely relevant to chicken producers and vaccine manufacturers is essential.

## Introduction

Poultry health and welfare is threatened by a number of pathogens and protozoan parasites of the genus *Eimeria* are among the most important. Infection with *Eimeria*, which invade and replicate within gut epithelial cells, can compromise chicken welfare and reduce productivity in both layer and broiler systems requiring relatively costly treatments. The cost of these interventions, combined with the losses caused by infection, are estimated to cost the global chicken industry in excess of £2 billion every year (1). *Eimeria* infection has also been demonstrated to exacerbate the outcome of exposure to other pathogens such as *Clostridium perfringens*, combining to cause necrotic enteritis (2).

Control of *Eimeria* in commercial chicken production relies on routine chemoprophylaxis (primarily broiler chickens) or vaccination using formulations of live virulent or attenuated *Eimeria* species (layer and breeder chickens). Mass administration of anticoccidial drugs has long been employed as a highly effective method of control, however drug resistance is widespread and public/legislative pressure demanding reduced use in livestock production is intensifying (3, 4). Classification of ionophores as antibiotics in markets such as the United States of America has reduced use further as demand for “no antibiotics, ever” food products grows, prompting more than 40% of producers to include vaccination in one or more production cycles per year (5). Unfortunately, existing vaccines are relatively expensive due to high production costs and are difficult to scale up, especially those that include attenuated parasite lines. In response, demand for cost-effective anticoccidial vaccines is greater than ever.

Seven species of *Eimeria* are recognized to infect chickens and these vary in their fecundity, pathogenicity and location of replication within the gut [reviewed in Chapman (6)]. Infection with each species induces a robust protective immune response against homologous challenge (7). However, the numbers of parasites and rounds of infection required to induce immunity that is sufficient to protect against disease varies depending on the infecting *Eimeria* species, dosing schedule and chicken breed (7–10). There is little or no immune protection against challenge infection with a heterologous species, and in some cases even infection with a different strain of the same species can escape immune protection (11, 12). Therefore, although immunization of chickens with live *Eimeria* oocysts is effective and has been the basis of live oral coccidiosis vaccines for almost 70 years, chickens must be vaccinated with oocysts from each *Eimeria* species in order to be fully protected. The requirement for a live vaccine to include controlled doses of oocysts for all pathogenic species of *Eimeria*, and in some cases multiple strains of *Eimeria maxima*, makes vaccine manufacture logistically demanding as all vaccine lines have to be propagated separately in chickens under stringent specific pathogen free conditions. Another important consideration is that fecal-oral recycling of vaccine parasites is required to generate levels of protective immunity that are sufficient to protect chickens against pathogenic challenge by most *Eimeria* species (13). A recombinant vaccine that is protective against multiple *Eimeria* species is therefore highly desirable and this should contain multiple antigens, derived from different parasite species and lifecycle stages.

Criteria to identify a “good” new vaccine are difficult to define and may depend on the stakeholder asked. Sterile immunity, defined as the ability to stop all oocyst production following challenge, might be regarded as the primary objective but current anticoccidial drugs and live vaccines do not meet this stringent requirement (14, 15). Indeed, achievement of such strong evolutionary pressure may select for vaccine resistance (13). Important measures of efficacy that should be considered include decreased parasite load or replication, lowering environmental oocyst occurrence and thus transmission, reduced clinical signs of disease, and improved performance. Important performance parameters include feed conversion efficiency, body growth rate, flock homogeneity, and/or egg production. A vaccine should also be easy to administer, and safe for the target- and overlapping populations. Stability and consistency between batches is of utmost importance. Onset and duration of immunity are also important although the relevance of these parameters vary depending on the target population; short lived broiler chickens require vaccines with rapid onset of immunity, but duration is of less importance, whilst the opposite is commonly true for layer chickens. In the absence of effective correlates of protection, assessment of novel vaccines to protect against coccidiosis can only be performed *in-vivo* (as discussed below), but the design of experimental vaccine challenge studies has been highly variable in the published literature. This lack of standardization results in difficulty evaluating new vaccines and makes comparison between studies challenging. As well as study design, the choice of parameters evaluated during a vaccine challenge is also important when evaluating novel vaccines, particularly for intracellular pathogens such as *Eimeria* where antibody response is not a reliable correlate of protection against disease (16). This review examines the challenges in design of vaccination studies for chicken coccidiosis and methods for evaluating vaccine efficacy.

## Current *Eimeria* Vaccines

The first vaccines against coccidia used live, wild-type, sporulated *E. tenella* oocysts and were initially marketed in the 1950's based on observations that administration of low doses of oocysts over a number of days induced protective immunity against homologous challenge. Over time these first generation vaccines were developed to incorporate further *Eimeria* species and have been widely utilized, particularly in North America (15). Methods for delivery of these live wild type vaccines have improved over the years; common methods include spraying live oocysts directly onto day of hatch chicks in a spray cabinet, although vaccines can also be delivered by spraying onto feed, in an edible gel or in drinking water (17). Nevertheless, uniform administration of oocysts is not always accomplished and recycling of live oocysts by reinfection from litter is required for the development of robust immunity within the flock (18). Recycling of oocysts is difficult in traditional layer cage setups, although the addition of floor coverings have been trialed and found to improve oocyst recycling (19). A significant risk in the use of these wild-type, and thus fully virulent, live vaccines is that large numbers of oocysts accumulate rapidly within chicken house litter resulting in high levels of exposure and clinical disease, even mortality, necessitating the use of anticoccidial drugs following vaccination (15).

Newer second generation anticoccidial vaccines frequently utilize oocysts derived from attenuated lines of *Eimeria* parasites, the majority of which have been selected for an abbreviated lifecycle termed “precocious development” [reviewed in Shirley and Bedrnik (20)]. Briefly, consistent selection during *in vivo* passage for those oocysts which are produced at the beginning of the patent period result in parasite lines with shorter prepatent periods, facilitated by fewer and/or smaller rounds of schizogony, and reduced total oocyst production (21). Precocious parasite lines exhibit reduced pathogenicity, but importantly retain their immunogenicity (22). The exception is an *E. tenella* line selected for growth in embryonic chicks (23, 24). As for wild type anticoccidial vaccines, attenuated vaccine formulations include a mixture of sporulated oocysts of different *Eimeria* species. Attenuated anticoccidial vaccines are also given orally, adopting strategies similar to those used for live wild-type vaccines.

Production of live wild-type and attenuated anticoccidial vaccines is limited by the necessity for *in-vivo* infection of chickens to produce oocysts as these cannot be produced *in vitro*. They are therefore costly and time-consuming to produce when compared to alternatives such as anticoccidial drugs, most notably for attenuated vaccine lines that are less productive than their wild-type equivalents. Attenuation of less fecund but highly pathogenic species such as *E. brunetti* and *E. necatrix* results in parasite lines with limited reproductive potential, making routine propagation challenging (25). Other limitations include the necessity for detailed quality control of each vaccine batch for efficacy that can only be achieved *in vivo*, as well as a short shelf life and the requirement for a cold chain without options for freezing or freeze drying. Such limitations in production of vaccinal parasite lines can restrict vaccine availability, encouraging demand for recombinant vaccines.

## Current Challenge Models Used in Anticoccidial Vaccine Development

A number of *Eimeria* antigens have been identified as valid anticoccidial vaccine candidates, but no recombinant vaccines are commercially available (13). Comparison of results from vaccination and challenge experiments using different, or even the same, antigens is challenging because of high variability in the study design of published vaccination trials (Table 1). Common differences include magnitude and timing of the challenge dose, vaccine formulation (expression system, antigen-adjuvant combination, and quantity of antigen), vaccine dosing schedule, chicken breed or genetic line, husbandry set up and the choice of parameters measured to assess protection. Each factor can be confounding, which makes it difficult to compare efficacy of different antigens between studies. Selection of chicken breed and vaccine schedules might also make it difficult to assess whether vaccines could work in a commercial setting with large numbers of outbred birds, where cost of dose per chicken and improvements in production parameters are of utmost importance. The use of common standard chicken lines that are widely available, such as Cobb500 or Ross 308 broilers, or Hyline layers, will encourage comparison between studies. However, the hybrid nature of these chicken lines can result in elevated levels of experimental variation. Using specific pathogen free and/or inbred chicken lines can reduce non-treatment variation and improve statistical validity.

**Table 1:** A summary of key published anticoccidial vaccination studies.

**Species (No. papers)**	**Formulation**	**Vaccine challenge study details**		**Outcome parameters measured**									
		**Breed** **L = layer** **B = Broiler**	**Challenge dose (oocyst) L = low, h = high**	**Oocyst output**	**Lesion score**	**Body weight gain**	**Feed conversion ratio**	**qPCR parasite replication**	**Histo-pathology**	**Survival rate**	**Anti-coccidial index (ACI)**	**Serum antibodies**	**Other immune parameters**
*E. acervulina* (22)	DNA 5/22	L 10/22	L 2/22 (5,000)	16	13	18	1	0	0	3	5	10	10
	rProtein 12/22	B 12/22	H 20/22 (<5000)										
	Other 5/22												
*E. brunetti* (2)	DNA 1/2	L 0/2	L 0/2	2	2	2	0	0	0	0	2	2	2
	rProtein 2/2	B 2/2	H 2/2 (100,000)										
	Other 0/2												
*E. maxima* (14)	DNA 3/14	L 3/14	L 5/14 (<1000)	12	6	11	1	0	1	4	3	10	9
	rProtein 7/14	B 5/14	H 9/14 (>1000)										
	Other 4/14	NS 3/14											
		Other 3/14											
*E. mitis* (1)	DNA 0/1	L 1/1	L 0/1	1	0	1	0	0	0	0	0	1	1
	rProtein 1/1	B 0/1	H 1/1 (100,000)										
	Other 0/1												
*E. tenella* (43)	DNA 13/43	L 16/43	L 2/43 (<1,000)	40	29	38	1	1	1	10	14	28	18
	rProtein 19/43	B 19/43	H 40/43 (>1,000)										
	Other 11/43	NS 2/43	NS 1/43										
		Other 6/43											
Multiple (8)	DNA 4/8	L 2/8	L 1/8 (<10,000)	6	5	5	0	0	0	2	4	4	3
	rProtein 5/8	B 5/8	H 7/8 (>10,000)										
	Other 0/8	NS 1/8											

*For further details see Supplementary Table 1. NS, not specified*.

## Ethical Considerations in Vaccine Challenge Studies

All studies using live animals must be well-designed and have animal welfare at the forefront. The three Rs principles should be considered; replace, reduce and refine animal usage (26). Where possible consider replacing the use of animals; novel antigens could be examined *in-vitro* using existing cell lines before moving to *in-vivo* work, although scope is very limited given the inability of *Eimeria* to complete its lifecycle efficiently *in-vitro*. Reducing the number of chickens used in a study is important whilst balancing the need for adequate statistical power to examine the effect of any novel vaccine (27). Reducing variability in response to infection and vaccination by optimal chicken breed selection and careful study design could reduce the number of individuals required to achieve a statistically meaningful result. Refinement of studies through careful consideration of animal housing and procedures should maximize animal welfare and improve results; unnecessary stress during housing and handling is likely to impact on vaccine response. For example, housing chickens in larger cages for oocyst counting experiments can reduce the efficacy of oocyst recovery, increasing variation between groups and compromising statistical validity. Previous studies have suggested a minimum of between five and 10 chickens per treatment group is required for assessment of differences between lesion scores due to variability in response to infection with *Eimeria*. (28), although the magnitude of the anticipated response should be used to inform an appropriate estimate of statistical power (e.g., 2-sample *t*-test method). Similarly, when using quantitative PCR (qPCR) to assess parasite genome copy numbers in the caeca of chickens infected with *E. tenella* a group size of a minimum of six birds was required during validation of the technique at 5 days post infection (27).

## Variables in Vaccine Challenge Study Design

### The Host

The choice of chicken breed or line used in an *Eimeria* vaccine challenge study is likely to be determined based on a number of criteria including relevance to the target chicken population, experimental setup, outcomes to be measured, and genetic background. Use of inbred chicken lines can reduce host variation in response to vaccination and *Eimeria* infection, offering opportunities to minimize the number of chickens needed to assess vaccination outcomes and streamline experimental design. However, vaccine responses and the consequences of challenge are likely to differ from the hybrid commercial chicken lines that most anticoccidial vaccines will be intended for. If using commercial layer or broiler chickens the choice of line is also important and again could be influenced by the target market of the vaccine. One advantage of examining vaccine response in layer chickens is that cages can more readily be used in place of floor pens. Keeping chickens in cages can permit more flexible study design, also offering better control of oocyst recycling and opportunities for fecal collection for accurate enumeration of oocyst excretion. However, if measuring production outcomes such as weight gain is desirable then the use of broiler chickens is likely to be advantageous over layers.

It has been demonstrated that some inbred chicken lines are more susceptible to *Eimeria* infection than others (29). A potential association between MHC haplotype and *Eimeria* infection outcome has been identified, although results of individual studies have been inconsistent, possibly influenced by the choice of chicken haplotypes examined, the infection model used, chicken infection histories, and measures of infection used to assess susceptibility/resistance (30–32). It is also likely that additional genes are involved in disease susceptibility/tolerance/resistance and this has been supported by some studies (33, 34). As well as influencing the outcome of challenge, it is also likely that the genetic background of the chicken will influence response to any novel vaccine being assessed. For example, there is evidence that MHC B locus haplotype may influence protection after challenge; lesion scores after challenge infection with *E. tenella* were reduced in B^5^B^5^ chickens vaccinated with a recombinant protein derived from oocysts, but this response was not seen in B^2^B^2^ chickens (35). However, in another study B^2^B^2^ chickens were found to be more protected than other lines by vaccination following challenge with *E. acervulina* (36). The choice of chicken line and genetic background therefore must be considered when designing vaccine challenge studies, and it may be necessary to test the same vaccines in different geographically distributed chicken breeds.

Rodent models are sometimes used to examine responses to coccidial infection but are unlikely to replace the need for work in chickens as not only do different species of *Eimeria* infect each host, but the immune system of rodents is very different compared to avians (37, 38). While there are many similarities between *Eimeria* species that infect birds or mammals and most replicate within enterocytes, fundamental differences include antigenic profiles, schizont size and number of rounds of schizogony (39, 40), all of which may impact on vaccination and immunological outcomes. The availability of immunological tools and reagents for rodent species continue to offer value to vaccine development, but there has been a notable expansion of resources for work with chickens (see https://www.immunologicaltoolbox.co.uk/ for details). Furthermore, knockout mice with well-characterized phenotypes are readily available and can be utilized to better understand immune responses to infection. Although the limitations of these knockout mice is becoming more apparent (41), some experiments are still easier to perform in these mice compared to chicken, and may shed light on pathogenicity. For example, infection of mice with *Eimeria vermiformis* shows some similarities to *E. maxima* infection of chickens in terms of immunogenicity, pathogenicity and the area of gut infected, and have been utilized as an infection model (42). Nevertheless, mechanisms of pathogenicity or immunogenicity in rodent models are not necessarily reproducible in avian species.

### The Parasite

The choice of *Eimeria* species used in vaccine development and validation is commonly based on regional priorities and identity of the candidate antigen(s) to be tested. The choice of *Eimeria* species influences study design. Variation in fecundity, pathogenicity, and immunogenicity among *Eimeria* species impacts on the outcome of vaccination and severity of disease, but also dictates the magnitude of challenge dose and study design. If a multivalent vaccine is developed to protect against multiple *Eimeria* species, efficacy against each species should initially be assessed in isolation with a separate challenge for each species to avoid confounding results. In some examples the choice of *Eimeria* strain/genotype is also important. Strain-specific antigenic variation has been noted to influence the outcome of vaccination for several species including *E. maxima* (11). The same strain or isolate used as template in production of a vaccine should also be employed as the challenge, ensuring antigenic as well as species homogeneity. Subsequent vaccine development should then include comparison with antigenically distinct strains to assess cross-protection. The choice of strain or isolate can vary between studies and regions, regularly prioritizing local parasites to promote local relevance. However, the use of multiple different strains, many of which may be antigenically distinct, precludes effective comparison between studies. Standardization using vaccines and challenges developed from reference strains such as Houghton (H) *Eimeria* strains (40) can improve opportunities for comparison.

Challenge doses should be determined empirically based on breed of chicken and species of *Eimeria* and should be tailored to the study design so that the desired outcome of vaccination can be measured reliably and at an appropriate time point post infection (e.g., reduction in lesion score or parasite replication). A lower challenge dose is more appropriate for measurements of parasite replication such as oocyst counts or quantitative PCR (qPCR) to measure *Eimeria* genome copies, as the linear dose dependent relationship between challenge dose and parasite replication eventually plateaus as the challenge dose is increased (27, 43). Any potential reduction in parasite replication as a result of your vaccination is thus difficult to interpret at higher challenge doses due to this biological phenomena. Higher challenge doses that induce at least moderate lesions and depress weight are more appropriate for assessment of reduction in pathogenicity by lesion scoring or for production measurements such as body weight gain. The age, quality and sporulation rate of oocysts used for challenge must also be considered; oocysts stored for a long time or inadequately (e.g., incorrect temperature) are likely to be less infectious. A reduction in pathogenicity *in-vivo* is usually observed when using oocysts older than 6 months of age and a reduction in replication can be detected from 3 months. Standardization of oocyst age at the time of dosing between experiments will promote comparison between studies, although it has been suggested that dose adjustment can be employed if using older cultures (44). Administering the challenge dose is usually performed by oral gavage of oocysts, either as a single dose or multiple doses to try to mimic natural infection. Infection from the environment can be initiated through re-use of litter or co-housing with infected individuals.

## Immunology and Pathogenesis of *Eimeria* Infection

Elucidating the effector cells and molecules involved in protective immunity induced by natural *Eimeria* infection and then eliciting either a similar response by vaccination or a different one, if this is protective, is the desired outcome of many novel vaccines. However, it has to be stressed that the definition of an effective vaccine may differ widely between stakeholder groups. Indeed, this can range from the “best case” scenario, the induction of sterile immunity to more realistic responses, such as reduction in shedding and/or reduction of clinical signs. However, it is clear that understanding these immune responses can offer valuable insight into vaccine development and efficacy. It is apparent that *Eimeria* sporozoites interact with both phagocytic and non-phagocytic cells within the gut, but this interaction does not always result in clearance of the parasite. Early studies suggested that *Eimeria* sporozoites are transported from the gut lumen to the crypt epithelium, where most species develop further, via the lamina propria in intraepithelial lymphocytes (45). Another study using monoclonal antibodies to stain duodenal sections infected with *E. acervulina* found sporozoites to be within CD8^+^ cells, macrophages and, to a lesser extent, CD4^+^ cells (46). The same study confirmed that both live and dead sporozoites were present within macrophages cultured *in-vitro*, suggesting that uptake into the macrophage might not result in parasite killing (46). However, subsequent publications concluded that the percentage of *E. tenella* sporozoites found within intraepithelial lymphocytes was relatively low (47). In this study, most sporozoites were found within enterocytes although the number located within lymphocytes was higher in the lamina propria (47). It was suggested that in birds immune following natural infection sporozoites remain within intraepithelial lymphocytes and do not penetrate the crypt epithelial cells (45), whereas others suggested that sporozoites remain within the lamina propria and do not enter the crypt epithelium or are inhibited from further development within the crypt epithelium (47). It is important to note that effector cells and molecules might vary between primary and secondary immune responses, and this is certainly true of *E. vermiformis* infection in mice (42). Therefore, these potential differences must be considered during development of vaccines and protocols for vaccine characterization.

*Eimeria* specific antibodies are generated during *Eimeria* infection, but they do not appear to be involved in controlling infection following oocyst challenge (7), and antibody levels are not correlated with disease susceptibility (16). Early studies demonstrated that bursectomised chickens, unable to generate an antibody response, were resistant to a secondary challenge with *E. tenella* suggesting that a serological response is not required for protective immunity (48). However, it has been demonstrated that sera from chickens recovering from infection may inhibit sporozoite invasion of chick kidney cells *in-vitro* (49). Lysis of sporozoites by immune sera and changes suggestive of agglutination have been demonstrated *in-vitro* but surface changes to sporozoites and to a lesser extent merozoites, visible by electron microscopy, were not detected when immune sera was heat inactivated suggesting that this action could be mediated by complement (50). Passive protection of chickens against *E. tenella* infection with a monoclonal antibody has also been demonstrated *in-vivo* (51). The role of secretory IgA was initially considered important in protecting against parasite invasion (49). Caecal contents from immunized chickens were demonstrated to reduce sporozoite invasion of a chick kidney cell line however parasite development was not associated with immunoglobulin level or neutralization of sporozoites (52). Similarly, sporozoite numbers were reduced in the intestinal lumen of previously challenged infected chickens but these sporozoites were still infective and anti-sporozoite gall bladder IgA was not associated with immunity to reinfection (53). Antibodies therefore do not appear to play an important role in protective immunity against *Eimeria* infection *in vivo* and cell mediated responses appear to be of more importance.

The role of T-lymphocytes in inducing a protective immune response to *Eimeria* infection has long been recognized in rodent models devoid of T-lymphocytes, as these were unable to control infection [reviewed by Rose (54)]. An initial study in athymic nude rats infected with *E. nieschulzi* demonstrated that these rats did not become immune to a second infection. In contrast, heterozygous (nu/+) rats were very resistant to reinfection, suggesting a role for T lymphocytes (55). Similarly chickens treated with cyclosporin A, which prevents proliferation of T-lymphocytes, were not immune to secondary infection and had increased oocyst output compared with untreated controls (56). Transfer of cell mediated immunity (CMI) is also possible; adoptive transfer of peripheral blood lymphocytes and splenocytes from infected, immunized chickens resulted in the transfer of antibody producing cells as well as CMI when given intravenously to uninfected chickens, as shown by reduced oocyst output following *E. maxima* infection (57). Lymphoproliferative responses of peripheral blood lymphocytes to sporozoite antigen appear to correlate with resistance to *E. maxima* in different inbred chicken lines in primary and secondary infection, whilst antibody responses are inversely correlated to resistance to *E. maxima* and *E. tenella* (58). In the mouse CD4^+^ T-cells, and to a lesser extent CD8^+^ T cells, appear to be more important in controlling primary *Eimeria* infection, whilst CD8^+^ T cells appear to be more important in secondary infections (59). However in chickens, several studies have suggested there are differences in the cell populations involved in controlling infection by different *Eimeria* species, in single or mixed species infections (60, 61). A more recent study showed increased proportions of cytotoxic CD8^+^(TCRγδ^−^CD8β^+^) cells following primary infection but the proportion of TCRγδ^−^CD8β^+^ in the caeca was only maintained for 3 days following secondary *E. tenella* infection before gradually declining (62). The same study demonstrated induced CD107a^+^ cell surface mobilization on cytotoxic TCRγδ^−^CD8β^+^cells, a marker of recent degranulation, following primary and secondary infection with *E. tenella* (62). In secondary *E. acervulina* infection the percentage of CD8^+^ intraepithelial cells is increased, and one study has demonstrated a greater proportion of CD8^+^ intraepithelial lymphocytes in resistant chickens 10 days post infection (63). Variability in T-cell responses might also be seen between different chicken lines, presumably due to genetic variation (64). The role of CD8^+^ cells in mediating resistance to secondary infection has been proposed to be killing of infected epithelial cells (65).

In early studies, cytokines produced by T-lymphocytes were presumed to mediate immune responses to *E. tenella* and *E. acervulina* infection in chickens injected with T-cell supernatants (66). The role of interferon-gamma (IFN-γ) was initially examined in *E. vermiformis* infected BALB/c mice, where blocking of endogenous IFN-γ by an IFN-γ specific monoclonal antibody *in-vivo* resulted in increased susceptibility to primary but not secondary infection, and increased oocyst production during primary infection compared with mice not injected with the antibody (67). The role for cytokines in primary but not secondary infection has been supported by a qPCR study examining cytokine transcripts following *E. maxima* infection that demonstrated upregulation of both Th1 and Th2 cytokines in primary but not secondary infection (68). Studies suggest that antigen specific IFN-γ responses from infected chicken splenic cells do not peak until days 20–25 post infection (69, 70), or even up to 35 days after primary infection in one study (71). During *E. tenella* infection the specific IFN- γ response in peripheral blood lymphocytes appears to occur earlier, around 8 days post infection, and may coincide with an increase in CD8^+^ cells in the peripheral blood (72). The role of other cytokines such as tumor necrosis factor (TNF) and interleukin (IL)-10 in controlling *Eimeria* infection has also been explored. TNF production by macrophages is upregulated following *Eimeria* infection, with peak production coinciding with the incidence of intestinal lesions, leading the authors to be speculate that release of this pro-inflammatory cytokine may enhance disease pathology (73). Another study found it to be upregulated in primary but not secondary infection with *E. tenella* and found differences between levels of TNF produced by different inbred chicken lines (74). IL-10 mRNA transcripts were found to be upregulated in the spleen and small intestine of susceptible chickens after infection with *E. maxima* (75). Similarly, in *E. tenella* infection IL-10 mRNA was upregulated in the caeca post-infection (76), although there does not appear to be a clear relationship between IL-10 expression during infection and caecal pathology (33). Other cytokines might be linked to *Eimeria*-associated pathology (77), but further work is needed to define the underlying mechanisms. It is clear than although many cytokines are upregulated during *Eimeria* infection and the primary response in particular, no single cytokine is a clear biomarker for disease susceptibility or progression; although this could be because of the heterogenous cell populations/tissues assayed.

The role of macrophages in controlling *Eimeria* infection has also been investigated. Assessment of phagocytosis of *Eimeria* sporozoites by chicken peritoneal macrophages *in-vitro* showed low levels for *E. tenella* and, to a lesser extent, *E. maxima* in macrophages taken from non-infected chickens. Phagocytosis by peritoneal macrophages from infected chickens was increased but varied depending on the stage and number of infections, and was enhanced by immune serum. However, it has to be stressed that peritoneal macrophages have been described to be functionally very different to tissue-resident macrophages in the gut (78), and may have a more immunomodulatory role rather than an antigen-presenting function (79). Quantification of macrophages during caecal infection with *E. tenella* showed higher absolute numbers in immune birds at 8 h post infection compared with naïve birds, however there was a greater increase in macrophages in naïve birds compared with immune birds (80). The same study also demonstrated that sporozoites were more likely to be located within or next to macrophages in the caeca of naïve birds compared with immune birds, where sporozoites were also more often located within or next to CD8^+^ cells (80). The role of macrophages is therefore somewhat unclear, although they are likely to play an important role as antigen presenting cells to CD4^+^ T cells in primary infection and might also act as effector cells, potentially destroying parasites through the production of reactive oxygen species. Natural killer (NK) cells, mononuclear cells with cytotoxic activity, might also play a role in *Eimeria* infection although this is still unclear and may be mediated through IFN- γ (81).

## Immunology and Relevance to *Eimeria* Vaccinology

It is clear that there is no single immune correlate of protection against *Eimeria* in the chicken. Circulating antibodies, IgY, IgM, and IgA, that are specific against *Eimeria* can be detected in infected chickens and ELISAs to measure serum antibody levels to *Eimeria* species in chickens have been developed (82, 83). Detection of antibodies raised against novel anticoccidial antigens post vaccination might be of some relevance in assessing antigenicity and can provide evidence of successful delivery. However, as serum antibody levels are not central to immune protection following *Eimeria* infection their relevance in assessment of vaccine efficacy is minimal. In natural immunity to *Eimeria* infection a cell mediated response is most important and presumably an efficacious vaccine will need to generate a similar response, ideally without inducing pathology. Measuring cell mediated responses to vaccination is more challenging than measuring serum antibodies, both technically and to interpret. Peripheral blood is the most easily acquired sample, although cell mediated responses in the periphery may differ compared with local responses in the gut. Examining cell mediated responses in organs such as the gut and spleen can only be achieved post mortem and, therefore, pre and post vaccine samples are not available for any individual chicken.

## Assessment of Protection in *Eimeria* Vaccinology

### Measures of Pathology

In the absence of clear identifiable immunological correlates of protection other relevant parameters must be used to assess vaccine efficacy. Reduction or absence of clinical disease can be assessed by observing individual chickens for signs of disease and more quantitatively by assessment of pathognomonic lesions in the gut. A standardized approach to scoring pathological lesions described by Johnson and Reid (28) is still widely utilized; a lesion scoring system from 0 to +4 was adopted for each *Eimeria* species with varying score criteria based on the infecting species and the nature of any lesions assessed during peak infection. Use of the lesion scoring system requires training from an experienced user or pathologist and can be subject to individual interpretation. It is therefore prudent to use the same individual or ideally multiple individuals to score all groups within each study and to consider that lesion scores might not be comparable between different studies examined by different individuals. Johnson and Reid (28) suggested that for some species such as *E. maxima*, lesion score does not appear to correlate with pathogenicity. Association between lesion scores and the clinical impact of coccidiosis appears more nuanced. For example, in one study chickens immunized with live oocysts and then challenged with a high *E. tenella* dose demonstrated high lesion scores but not the same reduction in body weight as unvaccinated challenged birds (84). Later studies confirmed that although some vaccinated chickens developed lesions, parasites did not appear to be associated with these lesions, possibly implicating the pro-inflammatory immune response (85).

### Measures of Parasite Replication

Comparison of *Eimeria* replication through measurement of parasite load or output in vaccinated, mock vaccinated and/or unvaccinated groups is a common method for assessing vaccine efficacy. Traditional measures include microscopy to count oocysts excreted per gram (OPG) of feces or persisting in litter, or total oocysts excreted per unit of time (e.g., per day), with vaccine efficacy calculated as the difference between vaccinated and unvaccinated groups or individuals. Oocyst counts from total fecal material collected per day, or throughout the parasite's patent period, are the gold standard, removing variation incurred by differences in fecal consistency. For reliable oocyst counts from litter, sampling should ideally be randomized, collecting litter from multiple pre-determined points to avoid unconscious bias and ensure representation from different individuals within the group, although this is obviously difficult to achieve for large groups of chickens (86, 87). Sampling to obtain oocyst counts for multiple species of *Eimeria* should be undertaken with caution due to a variance in fecundity between species, with more fecund species potentially masking the presence of less fecund species.

Quantitative PCR (qPCR) is a viable alternative to oocyst counts as a method to quantify the number of *Eimeria* genomes in a sample. Assays have been developed and validated for quantification of all seven *Eimeria* species that infect chickens, using genomic DNA extracted from purified oocysts and fecal samples (88). For *E. maxima*, qPCR has been applied to total genomic DNA extracted from jejunum/ileum tissues adjacent to Meckel's diverticulum to define replication dynamics in chicken lines with varying susceptibility to infection (89). For *E. tenella*, total genomic DNA extracted from caecal tissue was used as template to quantify parasite genome numbers, highlighting a significant association between genome number and size of the oocyst dose used to initiate the infection. This method has the advantage of requiring lower group numbers due to within reduced intra-group variation between chickens given the same oocyst challenge dose compared with fecal oocyst counts (27). DNA extracted directly from feces can be unsuitable due to the presence of inhibitors which can impact qPCR efficiency and therefore quantification accuracy, although an internal qPCR control can be used for standardization (90). Extraction from tissues or oocysts is preferable given lower occurrence of PCR inhibitors. Variation in oocyst sporulation rates can influence quantification by qPCR given that an unsporulated oocyst contains two genomes, while a fully sporulated oocyst contains eight. Some studies have utilized non-sporulated oocysts for DNA extraction to minimize the impact of sporulation rate on quantification (91). qPCR has also been applied to validate and standardize next generation sequencing analyses, such as deep sequencing or *Eimeria* species 18S rDNA PCR amplicons (92).

While microscopic quantification of oocyst numbers and qPCR are reproducible and highly sensitive techniques, parasite replication can be confounded by several parasite and host effects. Parasite effects include the reproductive potential of the parasite species and strain. Species such as *E. acervulina* are highly fecund, producing many millions of progeny oocysts per chicken. In contrast, species such as *E. maxima* are far less productive (93). Host effects include inherent genetic resistance or susceptibility, nutritional status and immune status as a consequence of prior exposure history (94, 95). Parasite replication can also be influenced by the crowding effect, a phenomenon characterized by reducing fecundity in response to increasing level of parasite challenge, although the mechanism remains unclear (43). Co-infection with other *Eimeria* species or other infectious agents might also influence oocyst output (96, 97).

### Measures of Productivity

Production measures are of utmost importance to the poultry industry when assessing any novel vaccine but can be difficult to measure and/or interpret in an experimental setting. Body weight gain is of primary significance during broiler production, but might only be impacted during severe infection where weight gain can be up to 60% lower than in uninfected chickens (98). However, experimental models of severe infection can be unpredictable and are undesirable from a welfare perspective. Williams and Catchpole (99) advocated the use of body weight gain up to 7 days post infection and feed conversion efficiency as measures of vaccine efficacy, and demonstrated the use of these criteria for the live attenuated vaccine Paracox™.

### Composite Measures of Efficacy

The anticoccidial index (ACI) has been developed as a method to evaluate resistance to anticoccidial medication (100, 101). McManus et al. (101) incorporated percentage survival, percentage relative weight gain, lesion score and oocyst count, combining pathological, parasitological and production traits. Jeffers (100) used the difference in weight between day 0 and day 7 post-infection and then subtracted the average score of fecal abnormality, where 0 equated to normal feces and 4 equated to no normal feces visible, divided by 10. Chapman and Shirley (102) used a modified version of the former protocol and assigned cut-offs for drug sensitivity; >160 = sensitive, partially resistant = 120–160 and <120 = resistant. Some vaccine studies have used ACI as a method to evaluate vaccine efficacy and assigned different cut-off scores for efficacy with some variation between studies; an ACI of <160 is generally considered to be ineffective (103–107). However, whether this adapted scoring method is overly simplistic to evaluate the efficacy and usefulness of vaccines in the field has yet to be determined.

Combining the information derived from measurement of multiple parameters and interpreting this information can be difficult as correlation is commonly poor between the different measurements of vaccine efficacy (99). This could be in part because parameters such as weight gain and feed conversion efficiency need to be assessed over a longer period of time than the 5–6 days post infection which is optimal for assessment of lesion score and parasite replication. Even for vaccines which are currently on the market and considered efficacious there is variability in vaccine response with some chickens developing pathological lesions, although lesion score is not necessarily associated with weight gain in these vaccinated birds (85). Furthermore, the variability in measurements of each parameter will be influenced by the population selected with commercial breeds more likely to show variability in response to infection and vaccination than more inbred lines. Studies have tried to model outcome parameters following *Eimeria* infection and fit them to an “infection index,” however this too can be difficult with models working more efficiently at certain inoculation doses. The time point of measuring infection outcomes is also likely to impact on correlation between parameters (108).

## Conclusions

Assessment of novel vaccines to protect against *Eimeria* infection in chickens is becoming more and more relevant with the discovery of new antigens for different *Eimeria* species and the development of new methods for delivery. The lack of a standardized approach to assessment of coccidial vaccine efficacy hampers comparison between studies, with different chicken breeds, challenge models, husbandry systems and outcomes being assessed. Ultimately, any new anticoccidial vaccine must be easily deliverable and relevant to the target market. While reductions in parasite replication and lesion score are important and might be considered optimal, producers in the majority broiler sector prioritize improvements in feed conversion efficiency and weight gain. As well as these considerations, future studies should adopt a methodical approach to animal study design and reporting, ideally conforming to the ARRIVE guidelines to maximize robustness, repeatability and transparency (109). Reporting of negative or inconclusive results must also be considered. A better understanding of response and variability to both infection and vaccination is imperative in informing future study design and minimizing animals used whilst maximizing outputs.

This manuscript has been assigned the reference PPS_01XXX by the RVC.

## Author Contributions

FS led preparation of this review, with input from DW, FT, and DB.

### Conflict of Interest

The authors declare that the research was conducted in the absence of any commercial or financial relationships that could be construed as a potential conflict of interest.
